# Regulation of protein translation initiation in response to ionizing radiation

**DOI:** 10.1186/1748-717X-8-35

**Published:** 2013-02-13

**Authors:** Donatella Trivigno, Laura Bornes, Stephan M Huber, Justine Rudner

**Affiliations:** 1 Department of Radiation Oncology, University Hospital of Tuebingen, Hoppe-Seyler-Str. 3, 72076, Tübingen, Germany; 2 Institute for Cell Biology, University Hospital Essen, Virchowstr. 173, 45147, Essen, Germany

**Keywords:** lonizing radiation, Protein translation, Eukaryotic initiation factor, Akt, mTOR, Apoptosis, Mcl-1

## Abstract

**Background:**

Proliferating tumor cells require continuous protein synthesis. *De novo* synthesis of most proteins is regulated through cap-dependent translation. Cellular stress such as ionizing radiation (IR) blocks cap-dependent translation resulting in shut-down of global protein translation which saves resources and energy needed for the stress response. At the same time, levels of proteins required for stress response are maintained or even increased. The study aimed to analyze the regulation of signaling pathways controlling protein translation in response to IR and the impact on Mcl-1, an anti-apoptotic and radioprotective protein, which levels rapidly decline upon IR.

**Methods:**

Protein levels and processing were analyzed by Western blot. The assembly of the translational pre-initiation complex was examined by Immunoprecipitation and pull-down experiments with 7-methyl GTP agarose. To analyze IR-induced cell death, dissipation of the mitochondrial membrane potential and DNA fragmentation were determined by flow cytometry. Protein levels of the different initiation factors were down-regulated using RNA interference approach.

**Results:**

IR induced caspase-dependent cleavage of the translational initiation factors eIF4G1, eIF3A, and eIF4B resulting in disassembly of the cap-dependent initiation complex. In addition, DAP5-dependent initiation complex that regulates IRES-dependent translation was disassembled in response to IR. Moreover, IR resulted in dephosphorylation of 4EBP1, an inhibitor of cap-dependent translation upstream of caspase activation. However, knock-down of eIF4G1, eIF4B, DAP5, or 4EBP1 did not affect IR-induced decline of the anti-apoptotic protein Mcl-1.

**Conclusion:**

Our data shows that cap-dependent translation is regulated at several levels in response to IR. However, the experiments indicate that IR-induced Mcl-1 decline is not a consequence of translational inhibition in Jurkat cells.

## Background

Cells need to replenish their protein pools of with every cell division. Therefore, protein synthesis is considerably up-regulated in proliferating tumor cells. The rate limiting step during protein synthesis is the initiation of translation which is regulated by several initiation factors (IF). They allow the recruitment of the initiator tRNA and mRNA to the 40S ribosomal subunit, recognition of the start codon AUG, and joining of the 40S and 60S ribosomal subunits leading to the formation of peptid bonds during protein elongation. The eucaryotic initiation factors eIF3, eIF4A, eIF4B, eIF4E, eIF4G1, eIF4H, and the poly A-binding protein (PABP) regulate the recruitment of mRNA to the initiation complex. eIF4E recognizes the 7-methyl GTP structure (cap structure) at the 5’ end of mRNA while PABP binds to the 3’ end of the mRNA. The helicase eIF4A, helped by eIF4B and eIF4H, unwinds the secondary mRNA structure at the 5’ end. The initiation factor eIF3, a multimeric complex of 13 different polypeptids (named eIF3A-M), interacts with the 43S ribosomal subunit that consist of 40S ribosomal subunit and the initiation factor eIF2 loaded with the initiator tRNA. Binding directly to the initiation factors eIF3, eIF4A, eIF4E, and to PABP, the scaffolding protein eIF-4G1/p220 coordinates the recruitment of the different factors into the initiation complex [1].

The formation of the initiation complex and the initiation of translation are further regulated by different signalling pathways which sense the optimal environmental growth conditions. Activation of the protein kinase B (PKB)/Akt pathway results in phosphorylation and activation of the mammalian target of rapamycin (mTOR). On the one hand, the protein kinase mTOR activates ribosomal protein S6 kinase (p70S6K) whose substrate is S6, a subunit of ribosomes [2]. When phosphorylated, S6 increases the translation of a subset of mRNAs that encode ribosomal proteins. On the other hand, mTOR controls cap-dependent translation through the translational inhibitor eIF4E-binding protein 1 (4EBP1). Upon phosphorylation by mTOR, 4EBP1 is not able to interact with eIF4E. Suboptimal growth conditions and environmental stress lead to inactivation of mTOR, dephosphorylation of 4EBP1 and an increased association of 4EBP1 with eIF4E preventing eIF4E from binding to eIF4G [3].

A quick shut down of global protein translation is important in response to cellular damage and environmental stress. It allows the cell to save resources and energy that is needed to repair the cellular defects. At the same time, it is important to maintain or even increase levels of proteins required for repair [4]. For this purpose, the cell can switch to cap-independent translation bypassing the cap-dependent translational inhibition. Around 10% of mRNAs contain an internal ribosome entry site (IRES) which allows a continuing translation of respective proteins under stress conditions [5]. IRES-dependent translation can be mediated by death associated protein 5 (DAP5 or p97), also known as eIF4G2 due to its homology to eIF4G1. In contrast to eIF4G1, DAP5 does not interact with eIF4E, but still binds to eIF3A and eIF4A. DAP5-dependent translational activity can be enhanced by caspase-dependent cleavage of DAP5 during apoptosis [6].

To secure survival under such unfavorable conditions, the cell must also maintain levels of protecting proteins. Myeloid cell leukemia sequence 1 (Mcl-1) belongs to the anti-apoptotic proteins of the Bcl-2 family that prevent apoptosis induction in response to many stress stimuli. Similar to the homologous proteins Bcl-2 and Bcl-xL, Mcl-1 is over-expressed in many tumors and associated with resistance to anti-neoplastic therapies such as ionizing radiation (IR). In many tumor cells, down-regulation of Mcl-1 is sufficient to induce apoptosis [7,8]. In contrast to Bcl-2 and Bcl-xL, Mcl-1 is an instable protein with a short half life time. Shut down of protein translation results in a rapid Mcl-1 decline and apoptosis induction [9,10].

So far, it has not yet been investigated whether radiation-induced down-regulation of the short-lived Mcl-1 is linked to inhibition of translation. Using Jurkat T lymphoma cells as a cell model, we examined protein translation and Mcl-1 levels in response to IR. Here, we could show that cap-dependent as well as cap-independent but DAP5-dependent translation were reduced after irradiation. Mcl-1 protein levels were regulated by an eIF4G1-dependent mechanism. However, RNAi knock-down of 4EBP1, eIF4B, eIF4G1, or DAP5 showed that neither cap-dependent nor DAP5-dependent translation affected radiation-induced Mcl-1 decline.

## Material and methods

### Inhibitors and antibodies

Pan-caspase inhibitor zVAD-fmk was purchased from Bachem (Bubendorf, Suisse), LY294002 was obtained from Cell Signaling (NEB, Frankfurt, Germany).

Following antibodies were used for Western blotting and immunoprecipitation: mouse-anti β-actin from Sigma (Deisenhofen, Germany), mouse-anti GAPDH from Abcam (Cambridge, UK), rabbit-anti Akt, phospho-Akt (S473), phospho-Akt (T308), mTOR, phospho-mTOR (S2448), phospho-mTOR (S2481), S6K, phospho-S6K (T389), 4EBP1, phospho-4EBP1 (T37/46), phospho-4EBP1 (T70), Mcl-1, eIF3A, eIF4A, eIF4B, eIF4E, eIF4G, phospho-eIF4G, and DAP5 from Cell Signaling (NEB, Frankfurt, Germany).

### Cells and cell culture

Jurkat E6 T-lymphoma cells were obtained from ATCC (Bethesda, Maryland, USA). Cells were grown in RPMI 1640 medium supplemented with 10% fetal calf serum (Gibco Life Technologies, Eggenstein, Germany) and maintained in a humidified incubator at 37°C and 5% CO_2_.

### Transfection with siRNA

Cells were cultured at a low density to ensure log phase growth. For transfection 3x10^6^ cells were resuspended in 300 μL RPMI-1640 without phenol red. Shortly before transfection, 4ebp1, eif4b, eif4g1, dap5, or non-targeting siRNA was added at indicated concentrations. The respective siRNA ON-TARGET SMARTpools and the siCONTROL NON-TARGETING pool siRNA were purchased from Dharmacon (Chicago, IL, USA). Cells were electroporated in a 4 mm cuvette in an EPI2500 square pulse electroporator (Fischer, Heidelberg, Germany) at 370 V for 9 msec. Immediately after transfection cells were resuspended in 6 mL pre-warmed medium and continued to be cultured as described above.

### Flow cytometric analysis

The mitochondrial membrane potential (ΔΨm) was analyzed using the ΔΨm specific dye TMRE (Molecular Probes, Mobitech, Goettingen, Germany). At the indicated time points, cells were stained for 30 min in PBS containing 25 nM TMRE. To measure DNA fragmentation, cells were incubated with PBS containing 0.1% Triton X-100 and 10 μg/ml propidium iodide. Cells were detected in channel 2 (488 nM excitation, 564–606 nm emission) employing a FACS Calibur flow cytometer (Becton Dickinson, Heidelberg, Germany) and analyzed with the FCS Express 3 software (De Novo Software, Los Angeles, CA, USA). Data show mean values +/− S.D. of 3 independent experiments. Statistical significance was calculated by a one-way ANOVA test using GraphPad Software (San Diego, CA, USA).

### Western blot analysis

Cells were lysed in 200 μL lysis buffer containing 50 mM HEPES pH 7.5, 150 mM NaCl, 1% Triton X-100, 1 mM EDTA, 10 mM sodium pyrophosphate, 10 mM NaF, 2 mM Na_3_VO_4_, 100 mM PMSF, 5 μg/ml Aprotinin, 5 μg/ml Leupeptin, and 3 μg/ml Pepstatin as described before [11]. Protein was separated by SDS-PAGE and transferred onto PVDF membranes (Roth, Karlsruhe, Germany). Membranes were blocked in TBS buffer containing 0.05% Tween 20 and 5% non-fat dry milk for 1 h at room temperature. The membrane was incubated overnight at 4°C with the respective primary antibodies. After repeated washings with TBS/Tween-20 (0.05%) the membranes were incubated with the secondary antibody for 1 h at room temperature before continuing to wash with TBS/Tween-20 (0.05%). Antibody binding was detected by enhanced chemoluminescence (ECL Western blotting analysis system, GE Healthcare/Amersham-Biosciences, Freiburg, Germany). Equal loading was verified by antibodies against β-actin or GAPDH. Where indicated protein levels were quantified by densitometry using ImageJ software (ImageJ 1.40 g NIH, USA). All Western blot experiments were repeated at least once.

### Cap pulldown

Cells were lysed as described for “Western blot analysis”. 60 μL of 7-methyl GTP-sepharose™ 4B beads (GE Healthcare/Amersham-Biosciences, Freiburg, Germany) were incubated with 250 μg of protein in 500 μl lysis buffer for 2 h at 4°C. Beads were washed trice with 500 μl lysis buffer. Proteins were eluted by boiling the beads for 5 min in 100 μL SDS sample buffer containing 2.5% β-mercaptoethanol. 30 μL were separated by SDS gel electrophoresis.

### Immunoprecipitation

Cells were lysed as described for “Western blot analysis” using 1% CHAPS instead of Triton X-100. The protein concentration was adjusted to 2 mg/mL. 1–2 μg antibody and 50 μL slurry Dynabeads® suspension (Invitrogen, Karlsruhe, Germany) were added to 750 μL lysate. After the precipitation for 3h at 4°C the beads were washed thrice with 300 μL lysis buffer containing 0.2% of the respective detergent. Proteins were eluted by boiling the beads for 5 min in 100 μL SDS sample buffer with β-mercaptoethanol. 30 μL were separated by SDS gel electrophoresis.

## Results

**Figure 1:**
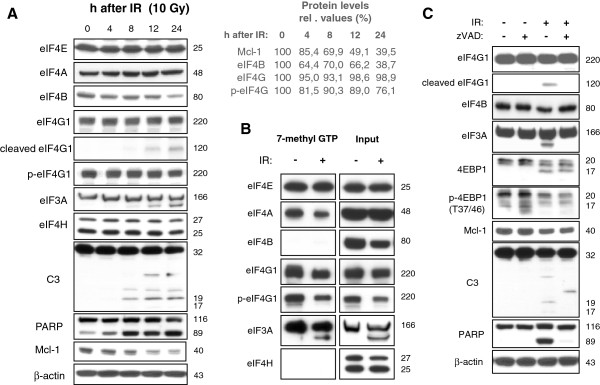
**IR induced caspase-dependent processing of eIF4G1, eIF4B, as well as eIF3A, and caused disassembly of the cap-dependent initiation complex. **Jurkat cells were irradiated with 10 Gy. (**A**) Lysates were made 4–24 h after IR. Cleavage of eIF4G1 and eIF3A, as well as eIF4B decline, were observed in response to IR. The cleavage coincided with processing of caspase-3 (C3) and the caspase-3 substrate PARP indicating caspase-3 activation. No IR-induced processing of eIF4E, eIF4A, and eIF4H was detected. Densitometric analysis shows relative protein levels normalized to the respective levels in non-irradiated cells (0h after IR). (**B**) 24 h after irradiation, cells were lysed and a pull-down assay was made using 7-methyl GTP agarose to mimic the cap structure of mRNAs. Whereas the recruitment of eIF4E to the cap structure remained unchanged, recruitment of eIF4A, eIF4G1, and eIF3A into the cap-dependent initiation complex was reduced after IR. (**C**) Jurkat cells were irradiated with 10 Gy and co-treated with 30 μM of caspase inhbitor zVAD or the respective amount of solvent. 24 h later, cells were lysed. Treatment with zVAD blocked IR-induced cleavage of eIF4G1, eIF3A, PARP, and the decline of eIF4B, as well as the processing of caspase-3 to the active p19 and p17 forms indicating that the processing of the three initiation factor occurred after caspase activation. However, caspase inhibitor zVAD did not block IR-induced 4EBP1 dephsophorylation on Mcl-1 reduction.

To analyze cap-dependent protein translation in response to IR, Jurkat T lymphoma cells were irradiated with 10 Gy. At indicated time, lysates were made and analyzed by Western blotting (Figure 1A). Caspase-3 and the known caspase-3 substrate, poly-(ADP-ribose) polymerase (PARP), were cleaved as early as 8 h after irradiation indicating the activation of the executioner caspase-3. While protein levels of eIF4A, eIF4E, and eIF4H did not change within 24 h after irradiation, cleavage of eIF4G1 and eIF3A was observed 12 h after irradiation. In addition, a continuous decline of eIF4B and the anti-apoptotic protein Mcl-1 was detected in response to irradiation.

The post-translational modifications of the initiation factors were correlated with disassembly of the initiation complex (Figure 1B). Using 7-methy-GTP agarose that imitates the cap structure in a pull-down assay, we could show that less eIF4G1, eIF4A, and eIF3A associated in the cap-binding initiation complex 24h after irradiation. In addition, dephosphorylation of eIF4G was observed 24 h after IR (Figure 1A). Decreased eIF4G phosphorylation correlated with less eIF4G binding to 7-methyl-GTP-recognizing complex (Figure 1B).

Caspase-3 and PARP were processed with kinetics similar to those of the initiation factors suggesting a correlation between caspase activation and cleavage of initiation factors (Figure 1A). To examine whether the cleavage of initiation factors was caspase-dependent, cells were treated with 30 μM pan-caspase inhibitor zVAD shortly before irradiation (Figure 1C). Western blots show caspase-3 (C3) and PARP processing that was inhibited by zVAD. Cleavage of eIF4G1, eIF3A, and eIF4B decline were also prevented by the caspase inhibitor suggesting that cleavage of eIF4G1 and eIF3A as well as eIF4B decline occurred after caspase activation. In contrast, radiation-induced 4EBP1 dephosphorylation and Mcl-1 decline were not inhibited by zVAD indicating a regulation independent of caspases. So far, our data suggest that the irradiation induced caspase-dependent cleavage of the initiation factors eIF4G1, eIF3A, and eIF4B, coinciding with a disassembly of the cap-dependent translation initiation complex.

**Figure 2:**
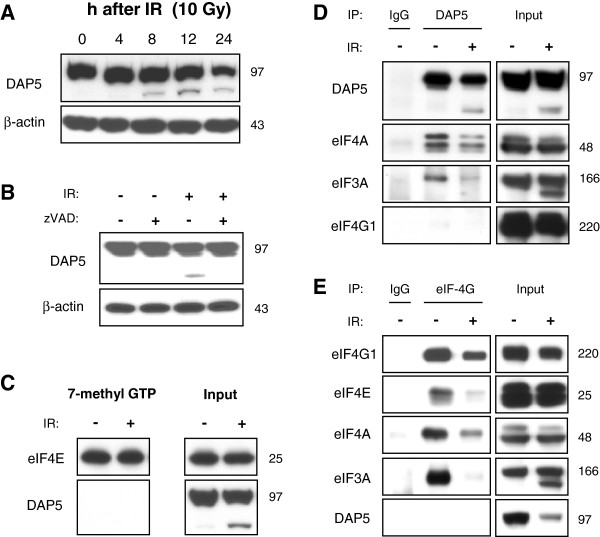
**IR induced disassembly of the eIF4G1-dependent as well as DAP5-dependent initiation complex. **Jurkat cells were irradiated with 10 Gy. (**A**) Lysates were made 4–24 h after IR. Cleavage of DAP5 was detected already 8 h after irradiation. (**B**) Jurkat cells were irradiated with 10 Gy and treated with 30 μM of caspase inhbitor zVAD or the respective amount of solvent. 24 h after irradiation, cells were lysed. Treatment with zVAD abrogated IR-induced cleavage of DAP5. (**C**) 24 h after IR with 10 Gy, cells were lysed and a pull-down assay was made using 7-methyl GTP agarose. DAP5 was not detected in the cap-dependent initiation complex. 24 h after IR with 10 Gy, cells were lysed and DAP5 (**D**), and eIF4G1 (**E**) were precipitated. (**D**) Less eIF4A and eIF3A co-precipitated with DAP5 (**D**) and eIF4G1 (**E**) after IR indicating disassembly of the DAP5-dependent and eIF4G1-dependent initiation complexes.

During radiation-induced apoptosis, a switch from cap-dependent to a cap-independent, but DAP5-dependent translation might occur. The DAP5-dependent translation is enhanced by proteolytic cleavage [12]. Indeed, cleavage fragment of DAP5 was detected in irradiated Jurkat (Figure 2A). DAP5 cleavage depended on caspase activation since treatment with caspase inhibitor zVAD prevented DAP5 processing (Figure 2B). DAP5 cannot interact with eIF4E and, therefore, was not detected in 7-methyl GTP-agarose pull-down experiments (Figure 2C). Similar to eIF4G1, DAP5 can interact with eIF3A and eIF4A [12]. We wanted to know whether there was a switch from cap-dependent, eIF4G1-dependent translation to a cap-independent, DAP5-dependent translation after irradiation. Such a switch would imply that the initiation factors eIF3 and eIF4A showed a decreased association with eIF4G1, but an increased association with DAP5. However, immunoprecipitation experiments revealed that eIF3A and eIF4A showed a reduced affinity to DAP5 (Figure 2D) and to eIF4G1 (Figure 2E) in response to irradiation indicating that both, eIF4G1-dependent and DAP5-dependent translation, were reduced upon IR.

**Figure 3:**
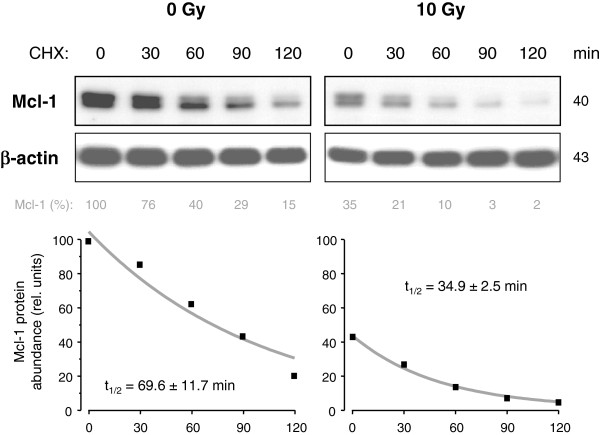
**Inhibition of protein translation results in rapid Mcl-1 decline. **Non-irradiated or, alternatively, 24 h after IR with 10 Gy, Jurkat cells were treated with 1 μM (CHX) for 0–120 min. Cells were lysed at respective time points. Mcl-1 levels were analyzed by Western blot (upper panel). Mcl-1 levels were quantified by densitometry and normalized to the Mcl-1 level of untreated non-irradiated control cells. The values were fitted and the half-life of Mcl-1 was calculated (lower panel).

**Figure 4:**
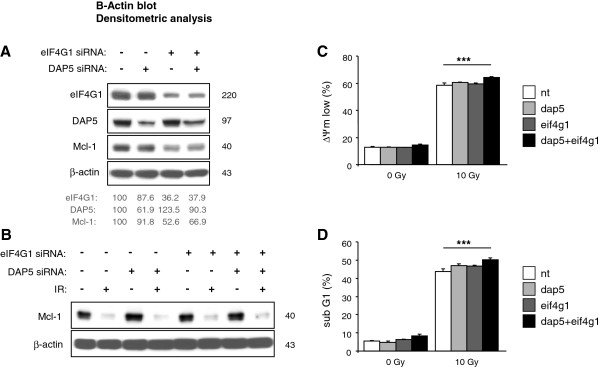
**Combined silencing of eIF4G1 and DAP5 accelerated radiation-induced apoptosis. **Jurkat cells were transfected with 500 nM eif4g1 siRNA or 500 nM dap5 siRNA alone, or with both, 500 nM eif4g1 and 500 nM dap5 siRNA. 1 μM non-targeting (nt) siRNA was electroporated into control cells. (**A**) 48 h later, cells were lysed and analyzed by Western blot. Protein levels were analyzed by densitometry and normalized to the respective levels in control cells transfected with non-targeting siRNA. Silencing of eIF4G1 resulted in Mcl-1 decrease suggesting that Mcl-1 translation in non-irradiated cells was eIF4G1-dependent but DAP5-independent. (**B-D**) 48 h after electroporation, cells were irradiated with 10 Gy. (**B**) Cells were lysed 24 h after IR. Silencing of eIF4G1 or DAP5 had no effect on radiation-induced Mcl-1 decline. Dissipation of the mitochondrial membrane potential (ΔΨm low, **C**) and DNA fragmentation (sub G1, **D**) were analyzed by flow cytometry 24 h and 48 h after IR, respectively. Slightly enhanced ΔΨm dissipation and DNA fragmentation was observed when both, eIF4G1 and DAP5, were silenced at the same time. Flow cytometric data shows mean values ± S.D. (n = 3), *** indicates p < 0.001.

**Figure 5:**
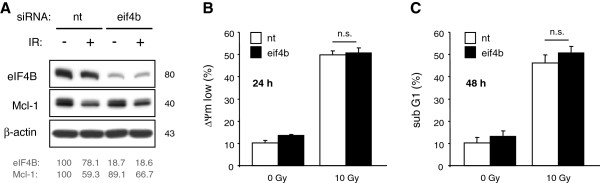
**Silencing of eIF4B did not change radiation-induced reduction of Mcl-1 and apoptosis induction. **Jurkat cells were transfected with 1μM eif4b siRNA by electroporation. 2 days later, cells were irradiated with 10 Gy. (**A**) Lysates were made 24 h after IR. eIF4B and Mcl-1 levels were analyzed by densitometry and normalized to the respective levels of non-irradiated cells transfected with non-targeting siRNA. Dissipation of mitochondrial membrane potential (ΔΨm low, **B**) and DNA fragmentation (sub G1, **C**) were analyzed by flow cytometry 24 h and 48 h after IR, respectively. Silencing of eIF4B had no impact on IR-induced Mcl-1 decline and apoptosis. Flow cytometric data shows mean values ± S.D. (n = 3), n.s. indicates no significance (p > 0.05).

To test the effect of translational inhibition on the short-lived Mcl-1, the translational inhibitor cycloheximide (CHX, 1 μM) was added to cell culture of non-irradiated cells or irradiated cells 24 h after irradiation with 10 Gy. Lysates were made 0 min, 30 min, 60 min, 90 min, and 120 min after adding CHX. Analysis of Mcl-1 protein levels showed a rapid decline of Mcl-1 following translational inhibition (Figure 3). Mcl-1 decline was faster in irradiated (t_1/2_ = 34.9 ± 2.5 min) than in non-irradiated (t_1/2_ = 69.6 ± 11.7 min) cells. To further analyze the putative role of cap-dependent and DAP5-dependent protein translation of Mcl-1 during radiation-induced apoptosis, eIF4G1 and DAP5 were down-regulated by siRNA. Western blot analysis showed that both proteins were successfully silenced 48 h after electroporation (Figure 4A). Silencing of eIF4G1, but not DAP5, resulted in a reduction of Mcl-1 protein levels indicating that eIF4G1-dependent translation rather than the DAP5-dependent translation regulated Mcl-1 translation in healthy Jurkat cells. However, radiation-induced Mcl-1decline was not affected by eIF4G1 and DAP5 siRNA (Figure 4B). In addition, flow cytometric analysis revealed that silencing of either eIF4G1 or DAP5 was not sufficient to significantly enhance ΔΨm dissipation (Figure 4C) and DNA fragmentation (Figure 4D). Only when both proteins were simultaneously down-regulated, a slight, but significant, increase of IR-induced ΔΨm dissipation and DNA fragmentation was observed. Our data suggests that eIF4G1-dependent translation regulated Mcl-1 protein levels, but the IR-induced down-regulation of Mcl-1 protein levels was independent of eIF4G1- or DAP5-dependent translation. In response to IR, eIF4B protein level was down-regulated in Jurkat cells. To test the involvement in IR-induced apoptosis, the initiation factor eIF4B was silenced by RNA interference (Figure 5). Silencing of eIF4B neither affected Mcl-1 decline (Figure 5A), nor ΔΨm dissipation (Figure 5B), nor DNA fragmentation (Figure 5C) in response to irradiation suggesting that eIF4B was irrelevant for the regulation of Mcl-1 levels in response to irradiation.

**Figure 6:**
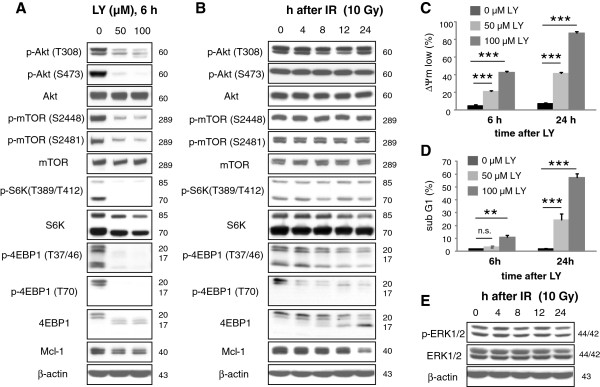
**IR induced 4EBP1 dephoshorylation independent of Akt/mTOR. **Jurkat cells were treated with 50 μM or 100 μM LY294002 (LY) (**A**) or irradiated with 10 Gy (**B**). Lysates were made 6 h after treatment with LY294002 or 4 h, 8 h, 12 h, and 24 h after IR. (**A**) LY294002 induced dephosphorylation of Akt on threonine 308 (T308) and serine 473 (S473) and on mTOR on serine 2448 and 2481 (S2448, S2481) indicating an inhibition of the Akt/mTOR pathway. Dephosphorylation of p70S6K on threonine 389 (T389) of p85S6K on threonine 412 (T412), and of 4EBP1 on threonine 37/46 (T37/46) and threonine 70 (T70) was also observed after treatment with LY294002. (**B**) IR did not affect phospho-Akt, phospho-mTOR, and phospho-S6K levels, but reduced phospho-4EBP1 levels as indicated by anti-phospho-4EBP1 antibodies and a shift of 4EBP1 from p20 to p17, suggesting that IR induced 4EBP1 dephoshphorylation independent of Akt/mTOR pathway. Reduction of Mcl-1 levels in response to LY294002 and IR correlated with 4EBP1 dephosphorylation. (**C, D**) Jurkat cells were treated with 50 μM or 100 μM LY294002. 6 h and 24 h later, mitochondrial dissipation (ΔΨm low, **C**) and DNA fragmentation (sub G1, **D**) were analyzed by flow cytometry. LY294002 induced mitochondrial dissipation and DNA fragmentation in a time- and concentration-dependent manner. (E) Jurkat cells were irradiated with 10 Gy. 4–24 h after irradiation, cells were lysed. Phospho-ERK1/2 and ERK levels were analyzed by western blot. No changes of phospho-ERK1/2 and ERK1/2 levels were observed in response to IR. Flow cytometric data shows mean values ± S.D. (n = 3), ** indicates p < 0.01, *** indicates p < 0.001, n.s. indicates no significance (p > 0.05).

**Figure 7:**
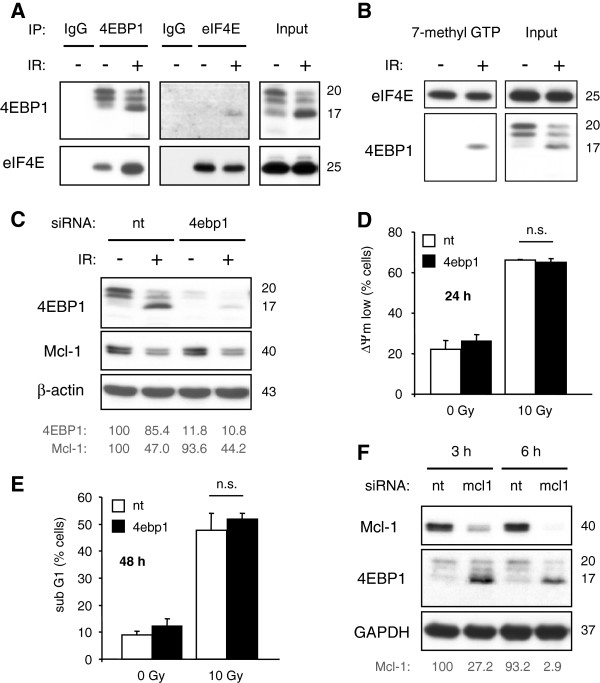
**Radiation-induced Mcl-1 decline and apoptosis was not affected by silencing of 4EBP1. **(**A, B**) Jurkat cells were irradiated with 10 Gy. (**A**) 24 h after IR cells were lysed and immunoprecipitation was performed using antibodies against 4EBP1 or eIF4E. As negative controls, isotype-matched antibodies (IgG) were used. Equal input was verified by western blot. Interaction of 4EBP1 with eIF4E was enhanced in irradiated cells. (**B**) 24 h after irradiation, cells were lysed and and a pull-down assay was made using 7-methyl GTP agarose. 4EBP1 with eIF4E bound to 7-methyl GTP cap. (**C-E**) Jurkat cells were transfected with 1 μM 4ebp1 siRNA or non-targeting (nt) siRNA by electroporation. 48 h later, cells were irradiated with 10 Gy. (**C**) 24 h after irradiation, cells were lysed. 4EBP1 and Mcl-1 protein levels were analyzed by densitometry and normalized to the respective levels in non-irradiated cells transfected with non-targeting siRNA. Silencing of 4EBP1 had hardly any effect on Mcl-1 protein levels. Dissipation of the mitochondrial membrane potential (ΔΨm low, **D**) and DNA fragmentation (sub G1, **E**) were analyzed by flow cytometry 24 h and 48 h after IR, respectively. (**F**) Jurkat cells were transfected with 250 nM mcl1 siRNA. Lysates were made 3 h and 6 h after electroporation. Mcl-1 protein levels were analyzed by densitometry and normalized to the Mcl-1 level in lysates made 3 h after transfection with non-targeting siRNA. Down-regulation of Mcl-1 by siRNA resulted in a rapid dephosphorylation of 4EBP1 which was indicated by the shift to faster migrating p17 band. Flow cytometric data show mean values ± S.D. (n = 3), n.s. indicates no significance (p > 0.05).

Further analysis of protein translation focused on signal transduction through the kinases Akt and mTOR. The Akt/mTOR pathway controls protein translation through activation of p70S6K and inactivation of the translational inhibitor 4EBP1. Abrogation of this pathway should result in translational inhibition and a quick down-regulation of the instable Mcl-1. To this end, Jurkat T lymphoma cells were incubated with the PI3K inhibitor LY294002 (50–100 μM for 6 h; Figure 6A). Western blot analysis showed that LY294002 reduced phospho-Akt and phospho-mTOR levels indicating an inhibition of both, Akt and mTOR. The mTOR substrates p70S6K and 4EBP1 were also dephosphorylated upon treatment with LY294002, indicating p70S6K inactivation and activation of 4EBP1. In addition, LY294002 reduced Mcl-1 levels and induced mitochondrial dissipation (Figure 6C) as well as DNA fragmentation (Figure 6D) in a concentration- and time-dependent manner. Together, the results strongly suggest that Mcl-1 protein levels and Jurkat cells survival were controlled by the Akt/mTOR pathway. To examine the regulation of protein translation in response to IR, cells were irradiated with 10 Gy. Lysates were made 4–24 h later (Figure 6B). In contrast to LY294002, irradiation affected neither phospho-Akt nor phospho-mTOR, nor phospho-p70S6K. Apart from Akt, the kinases ERK1/2 were suggested to control protein translation through S6K phosphorylation [13]. Phosphorylated ERK1/2 indicates kinase activity. Phospho-ERK1/2 levels were not changed in response to irradiation (Figure 6E) suggesting a contributory regulation of ribosomal translation by ERK1/2. In contrast, 4EBP1 was dephosphorylated in a time-dependent manner in response to IR. Concurrent with 4EBP1 dephosphorylation, Mcl-1 was down-regulated (Figure 6B). Furthermore, co-precipitation (Figure 7A) and pull-down experiments (Figure 7B) showed an increased association of 4EBP1 with cap-binding eIF4E. The results implicate a radiation-induced inhibition of cap-dependent translation which was independent of the Akt/mTOR and ERK1/2 pathways.

To analyze the extent of 4EBP1 on protein translation, especially on Mcl-1 translation, Jurkat cells were transfected with 4EBP1-targeting (4ebp1) or non-targeting (nt) siRNA. 48 h after transfection, cells were irradiated with 10 Gy and lysates were made 24 h post-irradiation. Down-regulation of 4EBP1 was verified by Western blot (Figure 7C). Dissipation of mitochondrial membrane potential (ΔΨm low, Figure 7D) and DNA fragmentation (sub G1, Figure 7E) were analyzed 24 h or 48 h after irradiation, respectively. In cells with low 4EBP1 levels, we expected an impaired translational inhibition and, therefore, a stabilization of Mcl-1 in response to IR. The failure to down-regulate Mcl-1 should have increased radioresistance and decreased radiation-induced apoptosis. However, 4EBP1 knock-down neither affected Mcl-1 levels (Figure 7C) nor radiation-induced ΔΨm dissipation (Figure 7D) and DNA fragmentation (Figure 7E). Thus, the decline of Mcl-1 did not depend on cap-dependent translational inhibition. Notably, transfection with siRNA targeting Mcl-1 (mcl1) caused a rapid loss of Mcl-1 3–6 h after electroporation that coincided with 4EBP1 dephosphorylation as detected by the shift from 20 kDa to 17 kDa in Western blot (Figure 7F). This result indicates that the dephosphorylation of 4EBP1 could occur after depletion of Mcl-1 levels.

## Discussion

Protein translation is frequently deregulated in tumor cells and accelerates tumorigenesis [14,15]. Many translational components are over-expressed in tumor cells. High levels of several eIF3 subunits were associated with malignant transformation, whereas eIF4B was connected to proliferation and survival [16,17]. Moreover, overexpression of eIF3A resulted in decreased sensitivity to cisplatin [18] whereas overexpression of eIF4G1 increased survival in irradiated breast cancer cells [19] indicating that individual components of the translational machinery might regulate cell survival in response to DNA damage induced by drugs or IR. Jurkat T lymphoma cells activated caspases in response to IR which resulted in the cleavage of several translational components, among others eIF4G1, eIF4B, and eIF3A. Proteolytic cleavage of eIF4G1 and eIF4B by caspase-3 during apoptosis induction was observed earlier [20,21]. Although caspase-dependent cleavage of eIF3A has not been described yet, another component of the initiation factor eIF3, eIF3j, is a known caspase-3 substrate [22,23]. Evidently, caspase-3-mediated cleavage of translation initiation factors is a general mechanism during apoptosis.

IR-induced cleavage of eIF4G1, eIF4B, eIF3A, and DAP5 coincided with disassembly of the cap-/eIF4G1-dependent and IRES-/DAP5-dependent initiation complex in Jurkat cells. Thus, a switch from cap-/eIF4G1-dependent to IRES-/DAP5-dependent translation, as suggested before [6,24], was very unlikely. We conclude that the caspase-dependent cleavage of the eIFs mentioned above resulted in reduced cap- and DAP5-dependent translational initiation. Moreover, the ability to cleave the eIFs might also be responsible for the high toxicity of Jurkat cells to IR. Interestingly, the polyclonal antibody used to detect eIF4G1 should also have recognized eIF4G3/eIF4GII, a protein of 175 kDa homologue to eIF4G1. The failure to detect eIF4G2 suggests that Jurkat cells do not express this eIF4G member.

In addition to caspase-dependent regulation, caspase-independent regulation of cap-dependent translation was observed in irradiated Jurkat cells. This mechanism involved dephosphorylation of 4EBP1 and enhanced association of this translational inhibitor with the cap-binding eIF4E. Previous studies have shown that 4EBP1 was also a central regulator of cap-dependent translation in irradiated breast cancer cells [14]. Moreover, high phospho-4EBP1 levels correlated with grade and malignancy in breast tumors [25]. Phosphorylation of 4EBP1 is regulated by the Akt/mTOR pathway [2,13]. Inhibition of the Akt/mTOR pathway reduces cell survival [26]. Blocking the pathway by LY294002 in Jurkat cells resulted in dephosphorylation of 4EBP1 and S6K, implying a translational inhibition through binding of 4EBP1 to eIF4E and inhibition of S6K. In addition, LY294002 caused a descrease of Mcl-1 level and apoptosis induction. Surprisingly, IR induced dephosphorylation of 4EBP1 although Akt and mTOR remained active, as the phosphorylation state of both kinases indicated. Thus, 4EBP1 dephosphorylation and Mcl-1 decline did not result from Akt/mTOR inhibition. Probably, IR enhanced phosphatase activity leading to increased dephosphorylation of 4EBP1. The phosphatase responsible for the process has not been identified, yet. However, the phosphatase might be regulated independent of caspase-activation, since 4EBP1 was dephosphorylated even in cells treated with the pan-caspase inhibitor zVAD-fmk.

Interestingly, treatment with calyculin A, the unspecific inhibitor of Ser/Thr protein phosphatase (PP) 1 and 2 family, resulted in an accumulation of a phosphorylated high molecular weight form of 4EBP1 in Jurkat cells [27]. Apparently, phosphorylation of 4EBP1 not only regulates its association with eIF4E but also targets the protein for poly-ubiquitylation and degradation via proteasome. However, we could not test the effect of calyculin A on IR-induced 4EBP1 dephosphorylation in Jurkat cells due to its rapid cell death induction (unpublished observation).

We focused our research on 4EBP1, one of three members of the 4EBP family. Since the phosphorylation sites are homologues, we expect a similar regulation of the other two related proteins. However, we examined neither the expression not the phosphorylation state of 4EBP2 and 4EBP3 in Jurkat cells. Thus, we cannot exclude a different regulation of the other 4EBP members leaving open the possibility that alternative regulation of protein translation by 4EBP2 and 4EBP3 occurred in irradiated cells. Apart from that, IR resulted in 4EBP1 dephosphorylation without affecting S6K in Jurkat cells suggesting an additional regulation of protein translation. A recent publication indicates that S6K is not dephosphorylated in response to Akt inhibitors when the extracellular regulated kinase (ERK) 1/2 pathway is activated [13]. A persistent ERK 1/2 activation probably contributed to the ongoing phosphorylation of S6K in irradiated Jurkat cells.

Inhibition of translation causes a rapid reduction of short-lived proteins. For example, c-Myc, cyclin D1 and Mcl-1 were quickly down-regulated in lymphoma cells when cap-dependent translation was blocked [28]. Accordingly, stimulation of cap-dependent translation by PP2A inhibitor okadaic acid or PP2A knock-down increased levels of c-Myc and Mcl-1 in human lung cancer cells [29]. Failure to reduce Mcl-1 levels resulted in enhanced survival and radioresistance [30].

We detected a rapid Mcl-1 decline in Jurkat cells treated with the translational inhibitor CHX. CHX-induced Mcl-1 decline was also observed in glioblastoma and cervical cancer cells [9]. Moreover, previous studies have shown that Jurkat cell survival depended on Mcl-1 expression [7,11]. Depletion of Mcl-1 by siRNA resulted in apoptosis induction. Mcl-1 protein levels might also be decreased by caspase-dependent cleavage [31,32]. However, inhibition of caspases did not prevent IR-induced down-regulation of Mcl-1 and 4EBP1 dephosphorylation in Jurkat cells suggesting a caspase-independent regulation of Mcl-1 levels in response to IR. The coinciding caspase-independent Mcl-1 decline and translational inhibition due to dephosphorylated 4EBP1 rather suggests that IR-induced inhibition of cap-dependent translation caused depletion of Mcl-1. Indeed, knock-down of eIF4G1 by siRNA decreased Mcl-1 levels in Jurkat cells but, surprisingly, did not affect IR-induced Mcl-1 decline. In addition, down-regulation of eIF4B or DAP5 by siRNA affected Mcl-1 protein level neither in non-irradiated nor in irradiated cells. Our results suggest that the IR-induced Mcl-1 decline was not caused by inhibition of cap-dependent or IRES/DAP5-dependent translation although Mcl-1 expression was translationally controlled by eIF4G1 in non-irradiated, healthy Jurkat cells. Furthermore, silencing of eIF4B, eIF4G1, or DAP5 had no effect or, in case of simultanous eIF4G1 and DAP5 silencing, only very modest effects on radiation-induced apoptosis leaving the question about the biological relevance of these radiation effects open.

Recently, further mechanisms controlling Mcl-1 stability have been published. Mcl-1 ubiquitin ligase E3 (Mule) and the Skip/Cullin/F-Box ligase complex containing β-transducin repeat-containing protein (β-TrCP) or FBW7 were able to ubiquitylate Mcl-1 and target the protein for proteosomal degradation [33-36]. In contrast, the deubiquitinase USP9x prevented Mcl-1 degradation and stabilized Mcl-1 levels by removing the poly-ubiquitin chain [37]. Thus, Mcl-1 levels might be lowered by enhanced ubiquitylation or reduced deubiquitylation. Indeed, the accelerated Mcl-1 degradation in irradiated cells shown in Figure 3 and a previous publication [11] supports a post-translational regulation of Mcl-1. Recently, we have demonstrated that USP9x was activated in a Jurkat subclone, but not in the parental cell line, in response to IR [11]. The USP9x activation resulted in enhanced Mcl-1 deubiquitination and an increased resistance to IR-induced apoptosis in this specific clone. It remains to be shown whether IR also activates Mcl-1-specific ubiquitin ligases to facilitate Mcl-1 ubiquitylation and proteasomal degradation in Jurkat cells.

## Conclusions

**Figure 8:**
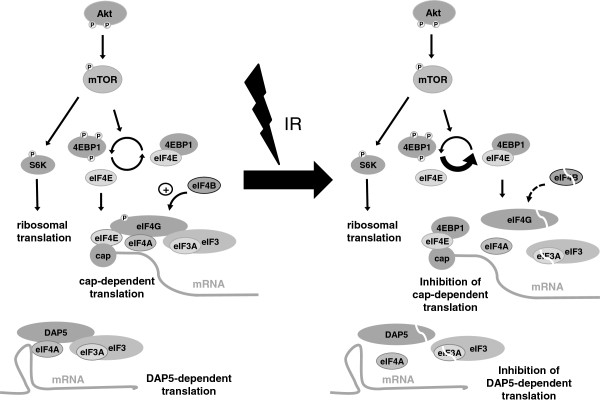
**Regulation of proteins translation in Jurkat cells. **Schema shows signaling pathway regulating cap-dependent translation in non-irradiated (left) and irradiated (right) cells. In non-irradiated Jurkat cells, the Akt signaling pathway is constitutively activated. Active Akt, indicated by two phosphorylation sites, phosphorylates and activates the kinase mTOR which in turn upregulates the ribosomal translation through phosphorylation of S6K. In addition, phosphorylation of 4EBP1 prevents the protein from binding to the initiation factor eIF4E and allows eIF4E to recruit eIF4G, eIF4A and the eIF3 complex containing the eIF3A subunit to the cap structure at the 5’ end of the mRNA. Thus assembled, the complex is able to initiate protein translation. eIF4B supports eIF4A thereby positively regulating protein translation. IR had no effect on Akt activity. Therefore, Akt and mTOR as well as S6K remain phosphorylated and activated 24 h after irradiation. In contrast, 4EBP1 is dephosphorylated in response to IR, probably due to an enhanced phosphatase activity. Hypophosphorylated 4EBP1 associates with eIF4E and prevents the recruitment of eIF4G to the cap structure. Furthermore, eIF4G, eIF3A, and eIF4B are cleaved downstream of caspase activation (indicated by the white line). As a consequence, the pre-initiation complex is disassembled and cap-dependent translation averted. In addition to eIF4G, eIF4A and the eIF3A complex associate with DAP5 in non-irradiated Jurkat cells suggesting a cap-independent protein translation through an alternative initiation site. In response to IR, DAP5 is cleaved in a caspase-dependent manner. The cleavage coincides with a disassembly of the DAP5-dependent initiation complex, probably resulting in reduced DAP5-dependent translation.

In summary, our results indicate that IR inhibited cap-/eIF4G1-dependent and DAP5-dependent translation in Jurkat cells. The mechanism leading to translational inhibition in response to IR involved caspase-dependent cleavage of eIF4G1, eIF4B, eIF3A, and DAP5 as well as caspase-independent dephosphorylation of 4EBP1. IR-induced decrease of phosphorylated 4EBP1 did not result from inhibition of Akt and mTOR but probably from increased phosphatase activity. The effects of IR on protein translation are summarized in Figure 8. We conclude that IR controled protein translation at different levels. However, decline of the short-lived, anti-apoptotic Mcl-1 in response to irradiation was not caused by inhibition of protein translation.

## Abbreviations

Mcl-1: Myeloid cell leukemia sequence 1; IR: Ionizing radiation; eIF: Eukaryotic initiation factor; DAP5: Death associated protein 5; 4EBP1: eIF4E binding protein 1; IRES: Internal ribosome entry site; mTOR: Mammalian target of rapamycin; ERK1/2: Extracellular regulated kinase 1/2; CHX: Cycloheximide.

## Competing interests

The authors declare that they have no competing interests.

## Authors’ contributions

JR designed the study. DT and JR, supported by LB and SMH, acquired, analyzed, and interpreted data. JR drafted and revised the manuscript. All authors read and approved the final manuscript.
